# Neurons in the monkey frontopolar cortex encode learning stage and goal during a fast learning task

**DOI:** 10.1371/journal.pbio.3002500

**Published:** 2024-02-16

**Authors:** Simon Nougaret, Lorenzo Ferrucci, Francesco Ceccarelli, Stefano Sacchetti, Danilo Benozzo, Valeria Fascianelli, Richard C. Saunders, Luc Renaud, Aldo Genovesio

**Affiliations:** 1 Department of Physiology and Pharmacology, Sapienza University of Rome, Rome, Italy; 2 PhD program in Behavioral Neuroscience, Sapienza University of Rome, Rome, Italy; 3 Laboratory of Neuropsychology, National Institute of Mental Health, Bethesda, Maryland, United States of America; 4 Institut de Neurosciences de la Timone, UMR7289, Centre National de la Recherche Scientifique and Aix-Marseille Université, Marseille, France; Oxford University, UNITED KINGDOM

## Abstract

The frontopolar cortex (FPC) is, to date, one of the least understood regions of the prefrontal cortex. The current understanding of its function suggests that it plays a role in the control of exploratory behaviors by coordinating the activities of other prefrontal cortex areas involved in decision-making and exploiting actions based on their outcomes. Based on this hypothesis, FPC would drive fast-learning processes through a valuation of the different alternatives. In our study, we used a modified version of a well-known paradigm, the object-in-place (OIP) task, to test this hypothesis in electrophysiology. This paradigm is designed to maximize learning, enabling monkeys to learn in one trial, which is an ability specifically impaired after a lesion of the FPC. We showed that FPC neurons presented an extremely specific pattern of activity by representing the learning stage, exploration versus exploitation, and the goal of the action. However, our results do not support the hypothesis that neurons in the frontal pole compute an evaluation of different alternatives. Indeed, the position of the chosen target was strongly encoded at its acquisition, but the position of the unchosen target was not. Once learned, this representation was also found at the problem presentation, suggesting a monitoring activity of the synthetic goal preceding its acquisition. Our results highlight important features of FPC neurons in fast-learning processes without confirming their role in the disengagement of cognitive control from the current goals.

## Abstract 2

Humans and animals learn by progressing from a first exploration phase to an exploitation phase. This study shows that neurons in the frontopolar cortex display a specific pattern of activity by representing the learning stage and the goal of the action.

## Introduction

In an ever-changing environment, we must face new situations and learn from them daily. This ability is especially developed in the most evolved species that are able to perform so-called one-trial learning, i.e., to determine in one trial which actions are more likely linked to a reward and to exploit this information. Recently, there has been an interest in the role played by the frontopolar cortex (FPC), the most rostral part of the prefrontal cortex in anthropoid primates, especially extended in humans [1–3], in these functions. Functional imaging studies in humans have suggested that this area is involved in a variety of cognitive skills [4–9], also providing evidence of an activation of the FPC during explorative behaviors [10–14]. Studies with FPC lesioned patients have not shown critical impairments in functions usually attributed to the frontal cortex, such as monitoring or working memory, but rather specific deficits that range across different cognitive domains, such as understanding and expressing emotions or managing multiple goals [15,16].

**Fig 1:**
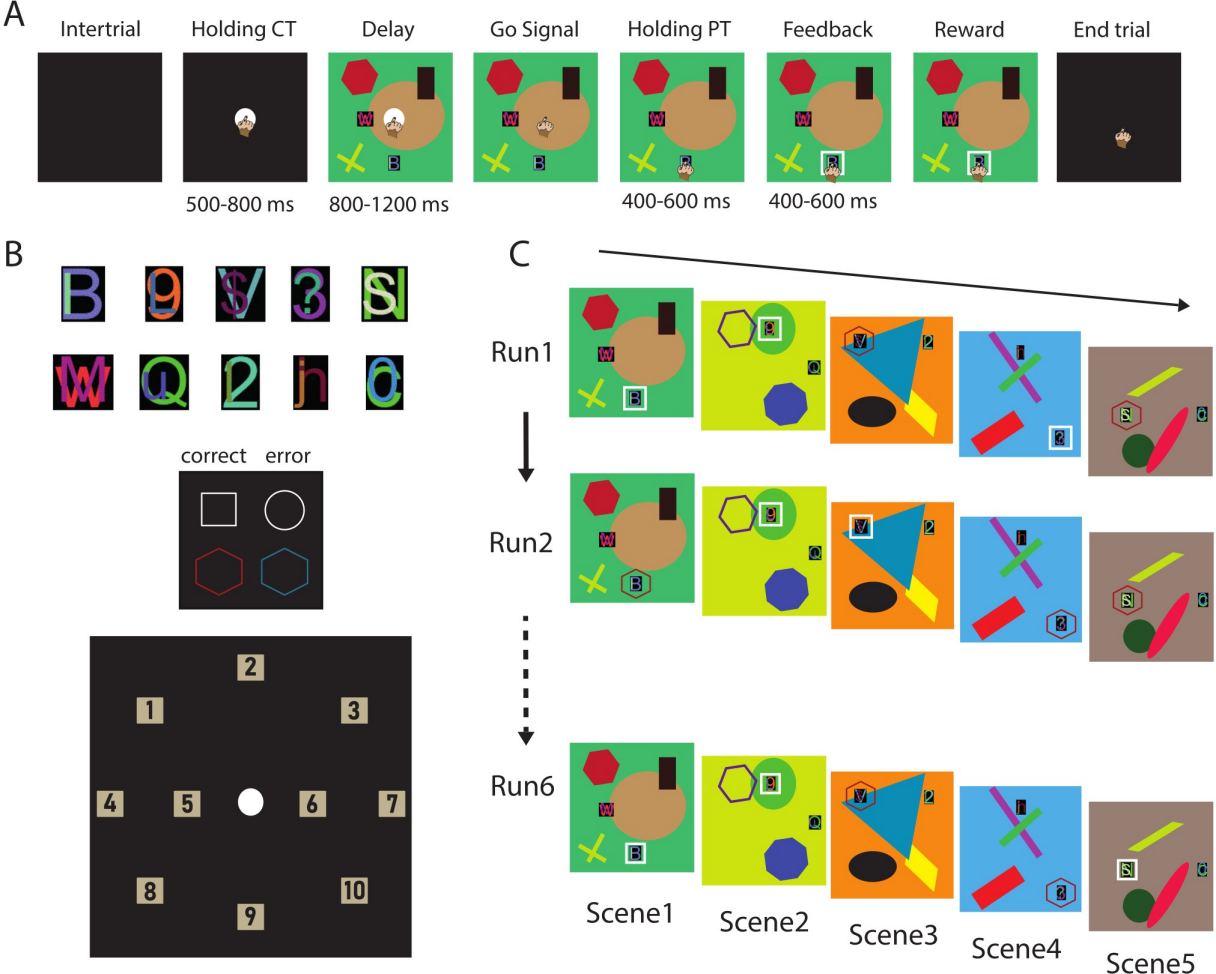
Object-in-place task. (A) Sequence of trial events and epochs. (B) Examples of peripheral target stimuli used in the different scenes (top); visual stimuli used as feedback around the chosen peripheral target (middle); all 10 possible spatial positions in which stimuli were presented on the screen (bottom). (C) Example of a block of trials. Five different scenes were presented in the same order for 6 consecutive runs (30 trials).

Phylogenetically, anthropoid primates are the only animals with a homologous area 10, which shares similar cytoarchitectonic features with humans [1,17,18]. Therefore, electrophysiological studies in macaque monkeys are providing extremely valuable information on the neuronal basis of FPC cognitive functions. Two seminal studies [19,20] reported that bilateral FPC lesions in macaques impaired rapid learning [20] but enhanced their ability to return to the main task after a distraction task [19]. These results led to the hypothesis that the FPC might be involved in the exploration of alternative options. Following the results of these pioneering lesion studies, we tested the hypothesis of a redistribution of cognitive resources to explain the fast-learning ability, in electrophysiology in behaving macaques. Precisely, the task was designed to untangle the coding of the goal of the action, the spatial position of the chosen target with one of the alternatives, the spatial position of the other available options. In the object-in-place task (OIP, Fig 1) [20–24], monkeys explored 5 distinct scenes, learned by trial and error which of 2 objects present in each scene was rewarded, and exploited the information gained to select the rewarded object in the next presentation of each scene. We confirm previous electrophysiological results [25,26] and report that the representation of the spatial position of the chosen target was encoded at the time of its acquisition. Moreover, we discovered 2 new properties of FPC neurons. First, they distinguished between the exploration and exploitation phases of the task, showing a specific tuning for the initial exploration of a scene. Second, and differently from our recent study [25], in which no learning was involved, we demonstrate that once the stimulus-action-outcome association was learned, since the second presentation of the problem, the chosen spatial position was represented earlier in the trial. Only in a learning context, the representation of the accomplished goal can be anticipated and emerged at the problem presentation when the monkey made its choice. Importantly, our results also demonstrate that only the chosen target was encoded and that the unchosen target was not. In line with the previous lesion studies [20], we confirm the role of the frontal pole in fast-learing processes; however, our results do not support the hypothesis that the frontal pole plays a role in the representation of the alternatives during a goal-directed behavior.

## Results

### One-trial learning

**Fig 2:**
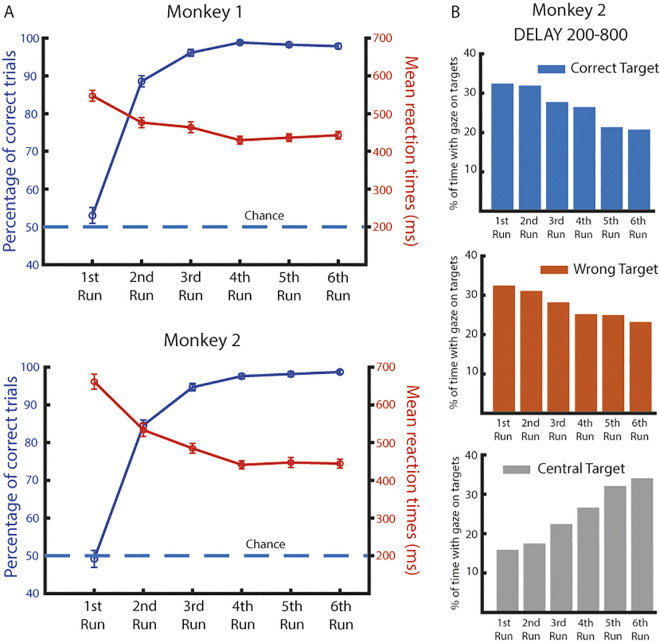
Behavior. (A) Behavioral performance and reaction times divided by run for each monkey. Dashed line represents the chance level for behavioral performance. Error bars represent the SEM. (B) Eye data for Monkey 2 during the Delay epoch (200 ms after the appearance of the scene until 800 ms after). Bars represent the average percentage of time across sessions spent observing the correct target, the wrong target, or the CT during each run. Source data are available in S1 Data.

Both animals had different background in the knowledge of the task. Monkey 1 had been trained extensively and his behavior was already reported in our precedent behavioral studies [23,24], whereas Monkey 2 reached a performance above chance level in the second run after only few days of training. Consequently, both monkeys learned to solve a given problem within a single trial, as seen by the high percentage of correct responses since the second run. As expected, choices were at chance level during run 1 and already above chance during run 2 (Fig 2A, blue curves; two-sample Kolmogorov–Smirnov test: first and second runs for Monkey 1, average 53.0% across 103 blocks, p = 0.9037 and average 88.8% across 103 blocks, p < 0.001, respectively; first and second runs for Monkey 2, average 49.2% across 109 blocks, p = 0.9955 and average 84.6% across 109 blocks, p < 0.001, respectively). Moreover, they were slower to make their choices in the first run (Fig 2A, red curves) and their RTs became lower over the runs (Monkey 1 showed a significantly higher reaction time during the first run compared to the remaining runs; Monkey 2 showed a decreasing reaction time until the third run, S1 Table). The eye movements were recorded from Monkey 2, and the distribution of the gaze’s position in space revealed a decrease from the first run to the sixth run in the amount of time in which the monkey explored the scene by looking at the targets. Conversely, moving forward in the runs, the time spent looking at the central target (CT) increased, to be more prepared for its disappearance and act faster (Fig 2B).

### Neurons in the FPC distinguish between exploration and exploitation phases of the task

**Fig 3:**
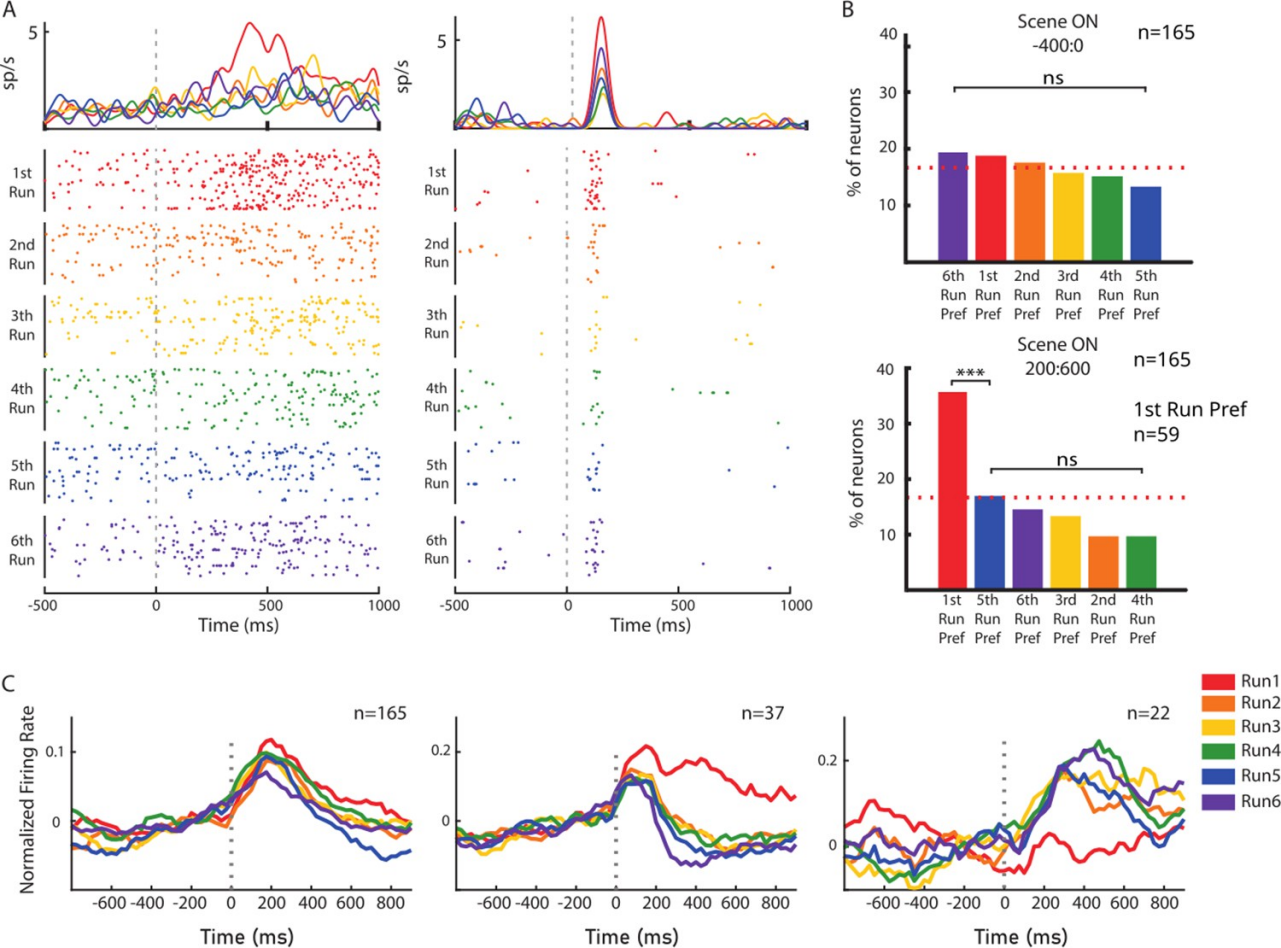
Single neuron preferences. (A) Raster plot of 2 single neurons (Monkey 1 on the left, Monkey 2 on the right) with a higher firing rate during the first run than in the remaining runs. Neural activity is aligned to the presentation of the scene (start of the Delay epoch). (B) Percentages of neurons with a preference for a specific run before (top) and after (bottom) the presentation of the scene. *** = p value < 0.001. ns = not significant. n = number of neurons. Percentages were calculated in the subpopulation of neurons responsive to the appearance of the scene. (C) Average response for each run of 3 subpopulations of neurons: all the neurons that respond to the appearance of the scene (left), and among these, neurons that prefer the first run with an increase in firing rate (center), and neurons that prefer the first run with a decrease in firing rate (right). Neural activity is aligned to the presentation of the scene (start of the Delay epoch), binned in a 200-ms window with a 25-ms step between each point, and normalized for each neuron by subtracting the mean activity and dividing by the standard deviation both calculated in a baseline period (−400 ms until the appearance of the scene). n = number of neurons. Source data are available in S1 Data.

Our study aimed to uncover the neural mechanisms underlying the FPC’s role in exploration and learning. To do this, we implanted bilaterally 96 or 48-channels UTAH arrays in the dorsal part of the frontopolar cortex (area 10), also defined as lateral frontal pole, in contrast to the medial and orbital parts of the area 10 (S1 Fig). We then focused on 2 main hypotheses: first, that the FPC’s neural activity would reflect the monkey’s level of experience with a given problem, with a greater difference in the neural representation of the scene during the initial exploration phase (first run) versus the later exploitation phase (second to sixth run). Second, that the FPC’s neural activity would encode the position of the chosen object, as it represents for each scene the solution of the problem, a crucial information for performing the task correctly. Both neural correlates were actually observed in our data. We focused our analysis on the delay period to capture these 2 processes. This period is of particular interest because it represented the time interval in which the features of the scene were associated with each problem. Moreover, it allows to observe the encoding of the chosen object’s position and to associate it with a learning process, differently than the monitoring of the performed action observed at the target acquisition and the feedback occurrence [25,26]. A high percentage of neurons modulated their activity after the appearance of the scene (165/399, 41.4% of the total recorded neurons; 72/159 [45.3%] for Monkey 1 and 93/240 [38.8%] for Monkey 2). In Fig 3A, the neuronal activity of 2 FPC neurons is shown, separated by runs, showing a higher activity at the appearance of the scene during the first run, before the solution could be known. The proportion of responsive neurons preferring the first run was significantly higher after the presentation of the scene (Fig 3B bottom; first preferred [first run] versus second preferred [fifth run] χ2 = 14.049, p = 0.0001782) but not during the baseline period (Fig 3B, top), ruling out that the neural response was just correlated to the passing of time throughout the 6 runs. We did not find a significant difference in the proportions of neurons preferring a specific run before the scene presentation (first preferred [sixth run] versus least preferred [fifth run] χ2 = 1.7935, p = 0.1805), highlighting the response specificity to the appearance of the scene. The average activity of these neuronal populations is presented in Fig 3C. Neurons that respond to the presentation of the scene are presented on the left (n = 165) and neurons that show a preference for the first run are presented in the middle (positive preference, n = 37) and on the right (negative preference, n = 22). Comparable results were found when considering the whole 399 neural population and applying a 1-way ANOVA on the same period with the run number as a factor (S2 Fig). Fourteen percent of neurons (56/399) were modulated and the majority of them (55%) preferred the first run. Moreover, we also found that the neuronal preference for the first run was irrespective of the differences in visual stimulus inputs (see Material and methods). We observed that a higher-than-chance number of problems preferred the first run in the 37 “positive cells” (334/1,058 of problems with a higher firing rate in the first run, 33%) and in the 22 “negative cells” (83/317 of problems with a lower firing rate in the first run, 26%).

**Fig 4:**
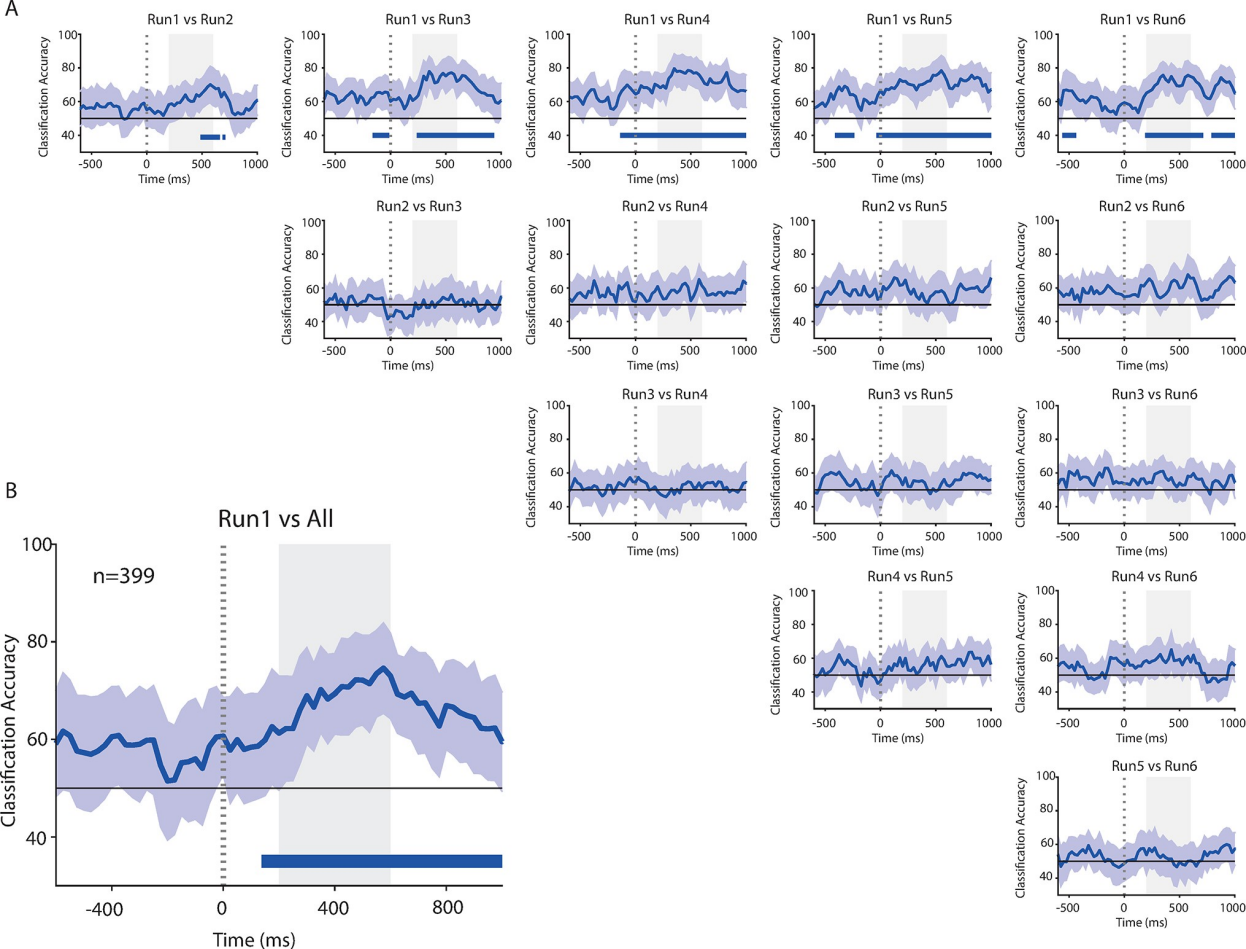
Explore vs. exploit. (A) Classification accuracy between all the possible pairs of runs (from 1 to 6) at the presentation of the scene. Blue bottom lines represent the periods during which classification accuracy is above chance level for at least 5 consecutive bins. (B) Classification accuracy between the first run and all the remaining runs collapsed together. n = number of neurons. Shaded gray areas represent the epoch during which the single neuron preferences were assessed (see Fig 3B). Source data are available in S1 Data.

To assess the contribution of the whole population of 399 neurons in fast-learning, we used a decoding procedure and classified the run performed by the monkey. The accuracy of this classification was compared to the one predicted by a null model and compared between each possible pair of runs (Fig 4A) and between the first run versus all the other runs together (Fig 4B). We found that the decoding accuracy between the first run and the subsequent runs (2 to 6) was above chance level (Fig 4A, upper line of plots). There was an increase in the decoding accuracy at the appearance of the scene for each 2-by-2 comparison involving the first run. Most importantly, the same analysis failed to report any significant difference in all other comparisons between runs 2 to 6. When the scene was presented in these latter cases, the neuronal activity was similar among the different runs and the decoding accuracy was at chance level. The activity in the frontal pole was no longer related to the run as information about the problem was learned and the system shifted from exploration to exploitation mode. Strikingly, very similar results were found in both monkeys (S3 and S4 Figs).

**Fig 5:**
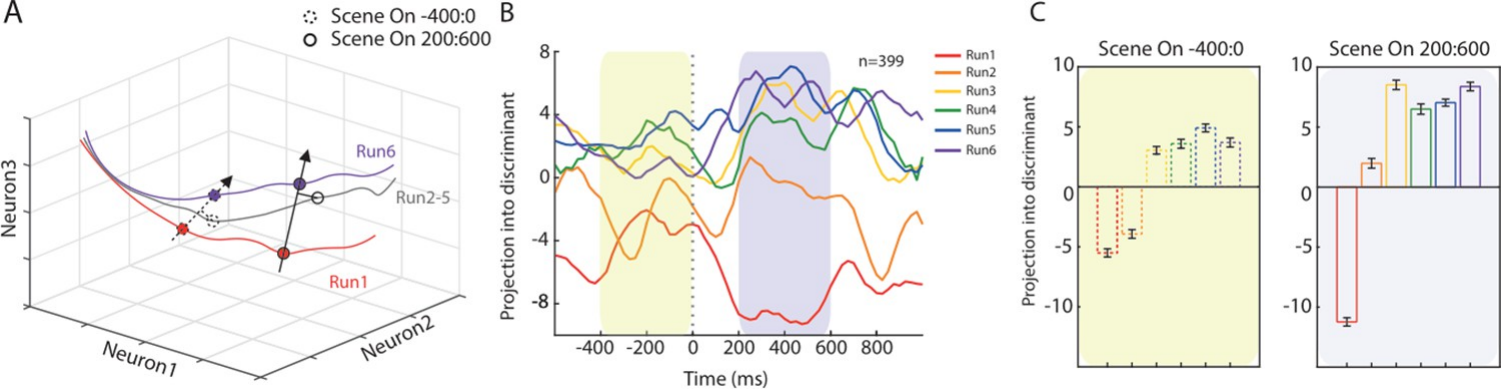
Difference among runs in the neural space. (A) Schematic illustration of neural trajectories in a 3D space where each axis represents the activity of 1 neuron. Circles represent different epochs. The vector originating from the circles on the gray line represents the projection for each run (from 2 to 5) into the discriminant. (B) Projection into the discriminant for each run, aligned to the presentation of the scene. (C) Mean value of the projections calculated in specific time windows, before the appearance of the scene (left, dashed circle in A) and after (right, solid circle in A). Source data are available in S1 Data.

To further shed light on the patterns of population activity across the different learning phases of the task, we investigated the similarities across the evolution of neural trajectories for the 6 runs. At each moment in time and during each experimental condition, neural activity can be represented in an N-dimensional space (i.e., a state space), where each dimension N is represented by the activity of one neuron at that specific moment [27,28]. As time moves forward, neural trajectories for different conditions can converge or diverge towards a specific point in the state space (Fig 5A). As previously shown with the decoding accuracy, we found that before and during the Delay epoch the distance between the first run and the remaining runs increased through time (Fig 5B), especially when comparing before and after the presentation of the scene (Fig 5C). In addition, we found that the second run lies in between the 2 well-separated clusters composed by the first run and runs 3, 4, 5, and 6, thus reflecting a distance that is not captured by the decoding procedure but that is consistent with the behavioral performance of both monkeys. Comparable results were found in both monkeys individually (S5 Fig). A control lower-level analysis on a more selected neural population (S2 Fig) already evidenced the intermediate position of the neural activity during the second run, highlighting the existence of a neural substrate of exploration at the single-cell level.

### Anticipated coding of the chosen position once learning occurred

**Fig 6:**
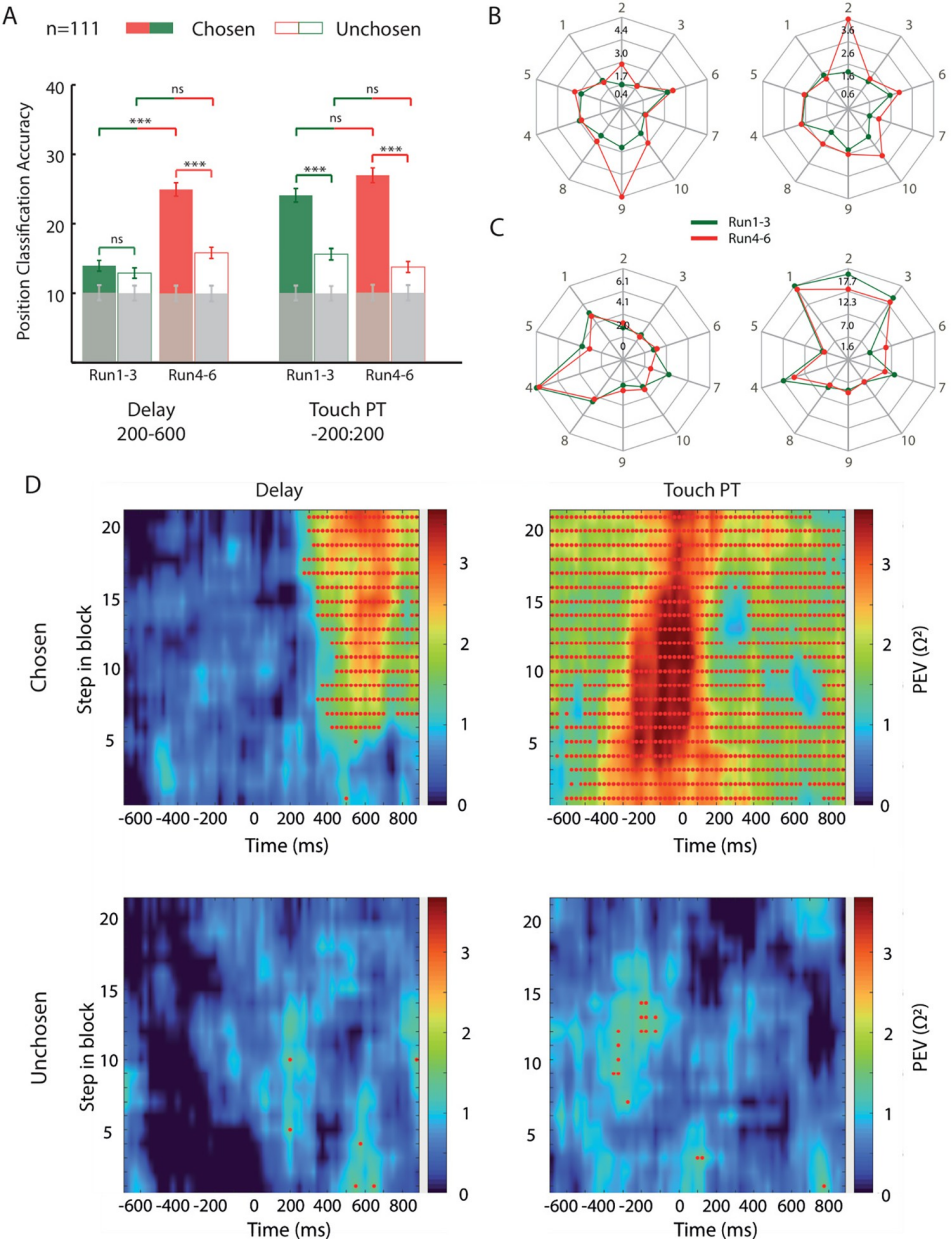
Effects of learning across runs. (A) Classification accuracy of the chosen and unchosen spatial positions during the Delay and Touch PT epochs, divided into trials of the first half of the block (from run 1 to run 3) and trials of the second half of the block (from run 4 to run 6). *** = η < 0.001. / = η > 0.05. n = number of neurons. Gray bars: decoding accuracy obtained by shuffling the labels. Vertical lines: mean standard deviation over resamples for each bar. (B) Examples of neurons that show spatial selectivity during the Delay epoch only in the first half of the block. Black numbers within the plot represent the average firing rate in the epoch of interest, while gray numbers outside the plot represent the chosen spatial position (see Fig 1B). (C) Examples of neurons that show spatial selectivity during the Touch PT epoch both in the first and second halves of the block. Markers as in (B). (D) Learning-related spatial selectivity in the population during the 2 epochs as a function of the number of trials in a block. Red dots indicate significant bins. Source data are available in S1 Data.

Then, we analyzed whether and how the solution of each problem was represented at the neural level. The same decoding procedure was applied, but the decoder was trained and tested on the position of the chosen or unchosen target instead of the run. Unfortunately, the number of trials for each position was very limited in each run, and the decoding procedure was not applicable individually for each run. For this reason, we separated the runs into 2 blocks to test for differences in the coding of the chosen or unchosen position after the solution of a specific problem had been thoroughly learned (runs 4 to 6) as opposed to when it had not yet (runs 1 to 3). During the Touch PT epoch, the decoding accuracy of the chosen position was identical between the first and second half of the runs (Fig 6A, right). On the other hand, during the Delay epoch, the difference was only significant in the final runs, when the monkey had learned the correct position from the previous runs (Fig 6A, left). Similar results were found for each monkey separately (S7 Fig). During the delay, the representation of the problem’s solution developed along the runs, along with the increase of the information learned. The unchosen position instead always showed a lower decoding accuracy compared to the chosen position, and did not show any significant difference between the first and the second half of the runs, neither during the Delay nor the Touch PT epoch. The low, but over chance, decoding accuracy found for the spatial position of the unchosen object was explainable by a task design bias (explained in S6 Fig) and the degree of overlap between the decoding of the spatial position of chosen and unchosen targets. Indeed, the positions of the correct and incorrect target pairs were not balanced inside the multiple sets of problems presented during a recording session. Consequently, some correct target positions were, by chance, mainly paired with some of the incorrect target positions in a given recording day (as exemplified in S6 Fig). Thus, a higher-than-chance decoding accuracy of the unchosen target position could have been driven by the frequent pairing with the chosen target position. The pattern of activity of the single neuron follows the dichotomy found between the 2 epochs. Fig 6B depicts 2 examples of neurons and their firing rate during the Delay period, sorted by the 10 spatial positions of the chosen target. Both neurons presented a spatial preference only in the second half of the blocks. Differently, Fig 6C represents 2 other neurons that have the same spatial preference equally in both halves during the Touch PT epoch. To better understand how spatial selectivity developed with learning, the variance in the firing rate that was accounted for by the chosen and the unchosen spatial positions (omega-squared) was assessed during the 2 epochs as a function of the number of trials in a complete block of 30 trials. Fig 6D shows no spatial selectivity in the early part of the block during the Delay period for the chosen position, which increases with the number of completed trials, while during the Touch PT epoch, the strength of the spatial selectivity is not affected by the number of completed trials, showing the highest tuning since the early part of the block. No effect was found for the unchosen position in both epochs. All the results were remarkably similar for the 2 monkeys (S7 and S8 Figs). The same analysis aligned at the occurrence of feedback (S9 Fig) shows the continuation of the response observed during the target acquisition (Touch PT period). The representation of the position of the chosen object appeared with learning during the delay, was stable and monitored during the action and tended to disappear with learning at the feedback. As a control analysis, we also investigated the representation of the spatial position of the chosen object at the single-cell level. In the first half of the blocks (runs 1 to 3), 14/111 neurons (12.6%, 8/77 for Monkey 1, 6/34 for Monkey 2) were selective for the spatial position of the chosen object against 20/111 neurons (18%, 10/77 for Monkey 1, 10/34 for Monkey 2) in the second part of the blocks (runs 4 to 6). Furthermore, we investigated whether the activity in the FPC was modulated during the delay of the second run by the switch versus non-switch of the previous choice. The trials of the second run were thus divided into 2 groups: the scenes where the correct object had been chosen in the first run and those where the wrong object had been chosen in the first run. In other words, trials were divided into those in which the same object as in the first run was to be chosen (previously chosen trials) and those in which the object not chosen in the first run was to be chosen (previously unchosen trials). The analysis was performed by selecting all trials and only the correct trials from the second run, and no significant coding was found in either case (S10 Fig). The switch to a previously unchosen alternative was not encoded during the second presentation of the problem.

## Discussion

Every choice leads to a consequence. Based on that consequence, one will decide whether to persist with that choice or shift toward other alternatives if it is presented again. After very few trials, humans and animals have the ability to progress from a first exploration phase to an exploitation phase by maximizing the benefit of the action. Explore–exploit decision-making is thought to be represented by a rostro-caudal gradient in the prefrontal cortex, with the FPC in charge of maintaining a level of exploration of the available options, whereas the caudal parts of the PFC compute the decision, allowing to exploit the current problem and lead the choice [2,29]. Human imaging studies also highlighted FPC activation during exploratory behaviors [10–12,30]. A recent study [13] using transcranial magnetic stimulation to suppress activity in the FPC during an explore–exploit task in humans found that this area was only activated when the exploration was directed, rather than random. A hypothesis suggests that activation during exploration could be interpreted as an evaluation of the available options, as alternatives to the main goal. In humans, the counterfactual choices, the unchosen options, selectively activate the lateral FPC, which has been identified as part of a prefrontal network involved in tracking specifically the relative unchosen probability and the counterfactual outcomes [8,31]. In the present study, we investigated the electrophysiological substrates of exploratory behavior through a fast-learning task, during which the performance was impaired in FPC lesioned monkeys. Then, we tested if this hypothesis could be supported at the neurophysiological level by finding a representation of the behavioral alternatives in the neural activity of the FPC.

In the object-in-place (OIP) task [21], designed to enable learning in a single trial, we found that monkeys learned very fast and reached a percentage of correct responses remarkably above chance since the second presentation of a problem, combined with a decrease in their reaction time. Their behaviors were composed of an initial exploration phase and subsequent exploitation phases. Our analysis revealed a first striking result, a high proportion of neurons that responded to the presentation of a scene had a stronger response during the first presentation, in the first run. By comparing the response to the scene before and after the scene presentation, we found that the increase in the neural response in the first run was specific to the Delay epoch. This response disappeared after the monkeys transitioned from an exploratory to an exploitative behavior, as they learned which object was the rewarded one (between the third and the sixth run, where the performance reached its plateau), and the scene lost its novelty. The intermediate results obtained in the second run suggest that the exploration phase may have lasted longer in the resolution of some problems. At the macroscopic scale, the FPC is part of a wider explore–exploit network that encompasses posterior prefrontal cortical structures, such as the dorsolateral prefrontal cortex, the orbitofrontal cortex, the anterior cingulate cortex, and subcortical limbic structures, such as the amygdala and ventral striatum [32–36]. Dorsal cortical structures can broadly be considered as an executive control network [2], controlling several aspects of action valuation and selection and allowing to transform goals into actions, whereas ventral prefrontal and cingulate cortical areas maintain the value of these objects [37]. However, this schematic representation has to be considered carefully. The ventromedial PFC, for example, is indeed involved in the representation of the value but specific paradigms revealed its importance in the shift between exploration and exploitation modes. In humans, Trudel and colleagues [38] dissociated accuracy and uncertainty effects on the choices of subjects and revealed that the polarity of uncertainty decision was driven by the mode (exploration or exploitation) the subject was. The exploitation of current goals has to be balanced with the possibility of choosing alternatives that are not grounded in an already valuable option. In the context of a fast-learning task, the unique pattern encountered during the first problem presentation may serve, based on the representation of the complex features of the problem, as a primer for choosing a new option and allocating attention when a new association needs to be built. It is, in a sense, a representation of the novelty that is encoded during the first presentation of a scene. It could be the trigger for the disruption of prefrontal dynamics observed during exploratory behavior in the posterior prefrontal cortex [39], supposed to support and maximize learning rate after exploration. We have previously considered the FPC important for learning the association between the cognitive processing generating what we defined as the synthetic goal and the response itself [26]. Synthetic goals differ from goals reached only following sensory instruction and are defined by the need to merge different sources of information, e.g., to apply a rule based on the memory of a previously performed action. Another interpretation is that the specific activity of the frontal pole during the first run reflects an evaluation of the monkey’s own ignorance; the animal knows that it does not know the solution of the problem. Indeed, Miyamoto and colleagues [40] reported in a MRI study in macaques that metacognition for non-experienced events is specifically localized in the area 10, and the inactivation of this area impaired selectively such events. Their whole-brain approach highlighted the specificity of area 10 in this process, unlike the neighboring area 9, for example, that represented experienced events in their study.

Our findings are consistent with earlier results [20] which showed that FPC lesioned monkeys were only impaired in fast learning but not general learning. Based on the literature, a hypothesis proposes that the impairment in one-trial learning after a frontal pole lesion could be due to a deficit in the representation of the alternatives from which monkeys also extract information [2]. In other words, the learning process could be slowed due to the loss of the ability to infer the value of alternatives when FPC is lesioned. To test this hypothesis, we tested if the positions of the chosen object and the unchosen object (the alternative) were represented along the different runs and could explain the difference found between exploration and exploitation. In different trials, each chosen target was presented with unchosen targets in multiple positions, allowing for the dissociation of their neural representation. In this task, once the general rule is learned, i.e., that there are 2 targets and only one is the correct one, during the execution of the first run it is possible to learn the solution of the problem regardless of whether the choice made was correct or incorrect. For this reason, the representation of both the chosen and unchosen object, especially during the first run, could help speed up the learning process drastically and represent a mechanism by which fast learning is processed in the FPC. However, our results argue against the hypothesis that neurons in the FPC are involved in the representation of alternative goals since the position of the unchosen object was not encoded at the time of choice, as opposed to the position of the chosen object. Furthermore, neurons in the FPC were not modulated when the monkeys switched to a previously unchosen alternative during the second run. The differentiation between the explorative and exploitative phases of the task, especially during the presentation of the scene, may represent the neural substrate through which the FPC supports rapid learning processes but not through the representation of the alternative. This discrepancy could be partially explained by the differences in the anatomical definition of the area 10 between human and nonhuman primates. The medial part of the frontopolar cortex (mFPC) can be matched with macaque area 10, whereas the lateral part of the human frontopolar cortex (lFPC) did not correspond to any prefrontal regions in macaques [41]. Functionally, lPFC in humans has been found to encode similar processes as the lateral prefrontal and the anterior cingulate cortex in macaque, i.e., representing counterfactual choices [42–44]. Moreover, studies in fMRI in macaques [42,44] brought a direct comparison of the involvement of each prefrontal brain area in the representation of the alternative option and failed to report such representation in the area 10. We can hypothesize that during the first run, the FPC neurons build different representations of the complex features of the scene, e.g., the combination between target and their context, useful to learn their association; however, it did not involve the representation of the position of the unchosen object as we initially predicted and it cannot be directly investigated with the current paradigm because scenes and objects are uniquely associated with spatial locations.

As opposed to the position of the unchosen object, we found that FPC neurons strongly encode the position of the chosen object. At the time of target acquisition, this information was present in both the first and second halves of the runs but was limited to the final half during the delay period. More precisely, the encoding of the position of the chosen object dynamically increased since the second run during the delay period. This finding is intriguing for 2 main reasons. First, to date, the 2 other electrophysiological studies investigating the FPC in monkeys [25,26] found spatial coding of the chosen position only at the time of or after target acquisition, interpreted as a re-representation of accomplished goals around feedback time. Indeed, neurons that represented the chosen position also showed an increase in correlation in the same time period compared to other neurons [45]. In the study of Tsujimoto and colleagues [46], a delay activity was present in the dorsolateral prefrontal cortex, indicating that the monkeys anticipated the choice, although it was not represented in the FPC. In our recent study [25], the spatial coding was not found during the delay period, and this is noteworthy in light of the fact that our results come from the same animals/implants used in that study. It has been proposed that the absence of spatial coding during the exploration phase reflects an undetermined state of the underlying neural network, a flexibility that allows the network to easily reshape itself to increase learning [39], thus representing a potential mechanism used by the FPC in rapid learning. Previous studies have shown that in decision-making tasks, the FPC’s role is primarily in retrospectively monitoring and evaluating decisions, as evidenced by the encoding of the spatial position of the chosen target only at the time of target acquisition [26]. In a fast-learning context, the representation of the target chosen in the FPC activity appeared not only in the same epoch as previously reported but also before, during the delay period, after the chosen target was learned for a given scene. It is important to note that the main difference between the current and past studies was the absence of learning. The spatial decision studied previously was based on the application of a strategy rather than the learning of the value of spatial stimuli. In previous studies [25,26], following a rule, a decision was made in every trial to choose the spatial position of the targets, and that position was not rigidly associated through learning with a stimulus. Instead, in the OIP, it was the spatial position of the target that was the key feature associated with a specific scene and an outcome. This information has been learned since the second run as an effect of the trials and errors exploration in the first run. The emergence of the coding of the position of the chosen object during the delay period cannot be interpreted as a working memory activity, as shown by a previous neurophysiological study that used a delay task as a control, or as movement planning, otherwise, it would have been present also during the first run. Our interpretation, based on the only neurophysiological studies available [25,26] is that the monitoring of the accomplished goal of the trial occurs even before its acquisition when the association between scene, objects, and spatial locations is formed. This effect is reminiscent of other types of anticipatory changes of the cell properties, as in the remapping of the visual receptive fields in the lateral intraparietal area, where receptive fields move in the new location even before eye movements [47]. The presence of a response during the delay period extends the known frontal pole neuronal properties. From our previous work [26], we have observed that when a behavior is externally cued, the neural response is restricted to the target acquisition, whereas when it involves a self-generation, the encoding lasts until feedback. In the present study, we discovered that an accomplished goal can be encoded even earlier, at the cue presentation likely as if it had already been accomplished. It cannot be ruled out that other factors also contributed to the learning process during the exploration phase. Since the objects presented in this version of the OIP task were always different across blocks to increase rapid learning, it is impossible to tell whether there was a specific encoding of the correct object. The first FPC electrophysiology study [26] failed to detect a representation of the visual objects when used as cues for the strategy leaving open the possibility that this was because the task goal was spatial. However, our later study [25], in which visual objects were used as a goal, also continued to find no coding of the object features in the FPC. Consequently, we are inclined to consider it unlikely that objects could be represented along with the spatial locations by the FPC neurons in the OIP task.

Our findings represent an important advancement in understanding the cognitive functions of the FPC, providing new evidence that is consistent with prior results and specific hypotheses, but not with others, about FPC functions obtained in human studies and lesion studies in nonhuman primates. However, new questions arose and should be addressed in the future. For example, since the OIP task is specifically designed to investigate rapid learning, it is yet to be determined how the FPC manages the transition between exploration and exploitation. It may be a categorical or binary switch from exploration to exploitation or a more gradual transition that follows a gradual reduction of uncertainty during learning (see [30]). Although different experimental paradigms are probably more appropriate to answer that question, the intermediate result obtained in the second run seems to support the second hypothesis. The inclusion of intermediate steps, obtained, for example, with the addition of multiple choice targets presented in the scene instead of a binary choice [36], would slow down learning, giving the possibility to disentangle novelty and uncertainty by reducing the former while maintaining the latter. An additional intriguing question remains how FPC neurons handle the explore–exploit trade-off, or the choice between exploring novel options with unknown value in search of new information and exploiting options with known values. Indeed, in our task, the monkeys were not given a choice between exploration and exploitation but were exposed to both phases successively. Introducing unknown objects when the associations are learned would lead the monkeys to actively choose to explore new behavioral options and would allow to compare FPC with other cortical and subcortical areas already investigated in monkeys [34,35,37,39] and humans [14] with such experimental paradigms.

Future studies should introduce an interfering event, such as a missed reward, to test whether the earlier coding of the goal is eliminated when confidence about the learned association is reduced. Moreover, we still do not know how long this earlier response lasts during a long learning process and when the problems become highly familiar. Therefore, it should be tested if such a response is transient only for the few successive presentations or if it persists even when the stimuli become very familiar to the monkey.

## Methods

### Ethics statement

Monkeys used in this study were monitored daily by the animal care staff or by the reseachers involved in the study. A veterinarian controlled regularly the general conditions of health and welfare of the animals. All surgical procedures were carried out under general anesthesia, using sterile techniques in approved surgery areas. Analgesics were used postoperatively to minimize discomfort or pain. Animal care, housing, and experimental procedures conformed to the European (Directive 210/63/EU) and Italian (DD.LL. 116/92 and 26/14) laws on the use of nonhuman primates in scientific research. The research protocol was approved by the Italian Health Ministry (Central Direction for the Veterinary Service, authorization n°1211/2015-PR).

### Subjects

Two monkeys (Macaca mulatta; Monkey 1: male, age 7 years, weight approximately 8 kg; Monkey 2: male, age 15 years, weight approximately 17 kg) were trained to perform a rapid-learning task before starting the recording sessions. The same monkeys were familiar with another social interactive task with a different rule [25]. The monkeys sat on a primate chair facing toward a touch-screen monitor (3M MicroTouch M1700SS 17” LCD touch monitor, 1,280 × 1,024 resolution) with their head fixed.

### Behavioral task

In the OIP task (described as performed in the current study in Ferrucci and colleagues [23]), geometrical visual cues were displayed on a touch-screen together with 2 objects between which the monkey had to choose. Two non-commercial software packages, CORTEX (NIMH, Bethesda, Maryland, United States of America, for Monkey 1) and MonkeyLogic (NIMH, Bethesda, Maryland, USA, for Monkey 2), were used to display images on the monitor, control a peristaltic pump delivering the reward, and record the behavioral responses of the monkeys. Eye positions during the execution of the task were monitored and recorded in all sessions for Monkey 2 through the ViewPoint Eye Tracker system (Arrington Research, Scottsdale, USA). The temporal course of the task is presented in Fig 1A. It started with the presentation of a CT that the monkey had to touch to make a visual scene appear. The scene was composed of 2 objects, one rewarded, one unrewarded, composed of 2 superimposed ASCII characters (Fig 1B, up), displayed in 2 out of 10 possible positions on the touch-screen (Fig 1B, down). These objects composed the problem the monkeys had to solve and were displaced on a colored background built from randomly created geometrical figures. Both objects were never presented in adjacent positions to avoid an overlap between the touching windows around each visual object. After the appearance of the scene, a delay period of 800 or 1,200 ms elapsed before the disappearance of the CT, which served as a “go” signal for the monkeys. The monkey had to choose and touch one of the objects presented in the scene. After 400 or 600 ms (Holding PT epoch), a visual feedback (randomly selected between different colors and shapes, Fig 1B, middle) was presented around the chosen object to indicate whether the choice made was correct or not. The feedback remained on the screen for 400 or 600 ms (Feedback epoch) and then disappeared; if the chosen object was correct, a reward (a drop of fruit juice) was delivered, while no reward was given in the case of wrong choices. If the monkey broke off its touch during any of the epochs before the presentation of the visual feedback, the trial was aborted and started again. The randomization process at multiple steps of the creation of a scene allowed us to present unique problems every block of 30 trials and therefore every recording day.

The monkeys encountered 5 successive problems presented for 6 runs (1 block; Fig 1C). During each run, the same 5 problems were presented in the same order until the sixth run was completed. Then, another block of 30 trials (5 problems*6 runs) started with the presentation of 5 new problems.

### Surgery and data collection

The surgical implantation and the data collection specificities were already described in [25]. The 2 monkeys were bilaterally implanted with high-density microelectrode chronic systems to record extracellular activity in the FPC (CerePort Utah Array, Blackrock Microsystems, Salt Lake City, Utah, USA; a 96-channel array in each hemisphere for Monkey 1; a 48-channel array in each hemisphere for Monkey 2). Since the signal was amplified and processed, the spike-sorting was performed using MountainSort, a fully automatic algorithm [48]. Details about filtering and spike-sorting parameters can be found in [25]. The dataset used for the following analysis was composed of 399 single neurons recorded during 24 days of the experiment, for a total of 212 blocks of 30 trials. From Monkey 1, 159 neurons (135 from the right hemisphere and 24 from the left hemisphere, 9 days, 103 blocks) were recorded. From Monkey 2, 240 neurons (169 from the right hemisphere and 71 from the left hemisphere, 15 days, 109 blocks) were recorded.

### Behavioral analysis

The percentage of correct trials was computed by dividing the number of correct responses performed in a run by the total number of complete trials performed in the same run in a given block, and then averaging across blocks. We compared the distribution of the percentages of correct trials across n blocks in the first and in the second run with a random distribution of performance across the same number of blocks. To do that, for each block, we sampled with replacement 5 values of 0 or 1 and obtained a sum (from 0 to 5) corresponding to the percentage of correct responses obtained by chance for a given block. Reaction times were defined as the times between the Go signal and the moment in which the monkey detached his hand from the CT and compared using a two-sample Kolmogorov–Smirnov test (p < 0.05).

### Single-unit analysis

We performed a first selection of neurons to identify the subpopulation modulated by the appearance of the scene, comparing the mean firing rate before the presentation of the scene (from 400 ms before to the appearance of the scene) and after its presentation (from 200 to 600 ms after) (Wilcoxon rank sum test, p < 0.05). The responsive neurons were then classified based on the preferred run as follows: for each neuron, the absolute difference between the mean firing rate of each run and the mean firing rate of all the remaining runs was computed, and the preference was assessed based on which run showed the highest difference, both before and after the scene presentation. Neurons with a preference for the first run were grouped based on the sign of this difference for the representation of population activities (Fig 3C, middle and left). Significant differences between proportions were assessed with a Pearson’s chi-squared test (p < 0.05). As a control analysis, a 1-way ANOVA with the run number as factor was performed on the same periods to select the “responding cells.” Two another 1-way ANOVA were perfomed to reveal the spatial selectivity of the chosen target and of the unchosen target. Each ANOVA had 10 possible positions as factor. Another control analysis was performed to analyze single-problem neural responses. The neural response to each single problem in each run (spike-count from 200 ms to 600 ms after the occurrence of the scene) was analyzed. Only neurons that responded (based on the Wilcoxon-rank sum test, see above) were considered. Positive and negative responsive cells were analyzed separately. We count the number of problems with a higher firing rate in each run. Only single problems that showed a higher/lower spike-count in a specific run compared to the other runs were considered (thus excluding problems that showed a shared maximum/minimum between 2 or more runs). We then compared to chance the proportion of problems with a higher or lower spike-count in each run.

### Decoding procedure

To evaluate the contribution of the entire recorded neuron population to rapid learning processes, we used a decoding procedure to discriminate 2 information: (1) the run performed by the monkey; and (2) the position of the chosen target. We used the Neural Decoding Toolbox [49]. For the decoding between runs, we followed this procedure: for each neuron, data were binned in the epoch of interest (200 ms bins, moving in steps of 25 ms (overlap 175 ms), from −600 ms to 1,000 ms from the appearance of the scene), and firing rate was normalized with a z-score transformation. For the run discrimination, trials were labeled based on the condition (run 1 to 6), and then divided into training and test splits adopting a k-splits procedure, where k represents the maximum number of available trials for each condition for the neuron with the lowest number of trials recorded per condition. A maximum correlation coefficient classifier was trained on the activity of all neurons in k-1 trials (k = 15; 399 neurons in total, 159 neurons for Monkey 1 and 240 neurons for Monkey 2), for each condition, and then tested on the activity of all neurons in the remaining split. When the correlation coefficient between training and testing trials under the same condition was higher than the correlation coefficient between training and testing trials under different conditions, a correct guess was made. This process was carried out k times, randomly sampling a testing trial, in order to calculate the average classification accuracy based on the number of accurate guesses. This procedure was repeated for n resamples (n = 50), where different k trials were selected from the recorded trials to be split into training and test sets. The final classification accuracy is represented by the mean value obtained across different resamples. Then, we repeated the entire process while randomly shuffling the condition labels to obtain a null distribution and test the significance of the average classification accuracy. The same procedure was adopted to discriminate previously chosen versus previously unchosen trials in the second run depicted in S10 Fig (200 ms bins, moving in steps of 25 ms (overlap 175 ms), from −600 ms to 1,000 ms from the appearance of the scene, k = 23, 310 neurons in total for all trials and 275 neurons in total for correct trials only). We followed the same procedure with minor changes for the chosen and the unchosen spatial position discrimination. Since there were not enough trials for each position and run combination, we divided the trials into 2 groups: trials from the first half of each block (run 1 to run 3) and trials from the second half of each block (run 4 to run 6). Within these blocks, trials were labeled depending on the chosen position (position from 1 to 10) and neural activity was binned in a unique bin of interest of 400 ms for the Delay epoch (from 200 ms after the appearance of the scene to 600 ms after) and for the Touch PT epoch (from 200 ms before the touch of the PT to 200 ms after). We repeated the decoding procedure selecting only those neurons with at least 5 trials for each position, chosen and unchosen, in each half of the block (k = 5; 111 neurons in total, 77 neurons for Monkey 1 and 34 neurons for Monkey 2), repeating the process k times for n resample run (n = 50) and then again for 1,000 times to gain statistical power and obtain a distribution of classification accuracies. The same procedure was repeated shuffling the condition labels to obtain a null distribution. Significant differences were assessed by computing the proportion of the overlapping area between the probability density functions of 2 distributions, defined by an overlapping index (η) [50]. η index indicating an overlap higher than 5% was considered not significant.

### Distances in the state space

To further investigate the similarities across different runs in the whole population of recorded neurons, we constructed a linear discriminant to represent the difference in neuronal activity at the population level between runs 6 and 1. We subsequently projected the activity of the 6 runs into the discriminant, following the procedure adopted in a similar learning task [36]. In brief, for each time bin, we divided the trials into 2 groups of equal size (train trials and test trials). For each neuron, we computed the mean firing rate in training and test trials in every run and the standard deviation in training trials to construct 3 matrices (M^train^, M^test^, and S^train^) of shapes n x r, where n is the number of neurons (399) and r is the number of runs (6). The activity in M^test^ was normalized for each neuron by subtracting the mean between the firing rate of training trials of the first and sixth runs, and dividing by the mean of the standard deviations calculated on the same runs, using the following formula to obtain the normalized matrix N^test^:

$${\forall i,j\;N}_{i,j}^{test}=\frac{M_{i,j}^{test}-\mu (M_{i,1}^{train},M_{i,6}^{train})}{\mu (S_{i,1}^{train},S_{i,6}^{train})}.$$

Where i,j represent the i^th^ neuron in the j^th^ condition (run). The same formula was applied to M^train^ to obtain the normalized matrix N^train^:

$${\forall i,j\;N}_{i,j}^{train}=\frac{M_{i,j}^{train}-\mu (M_{i,1}^{train},M_{i,6}^{train})}{\mu (S_{i,1}^{train},S_{i,6}^{train})}.$$

The linear discriminant V was obtained by subtracting the normalized vector obtained from the normalized training trials of the first run to the one obtained from the normalized training trials of the sixth run:

$$\forall i\;V_{i}=N_{i,6}^{train}-N_{i,1}^{train}.$$

The projections P into the discriminant is the vector whose i^th^ entry is the dot product between V and the i^th^ columns of N^test^ (6 columns, one for each run):

$$P=V^{T}N^{test}.$$

The whole procedure was repeated 100 times for each bin, using different training and test trial splits. This analysis was performed in 200 ms sliding windows (overlap 25 ms) around the appearance of the scene and on 2 different 400 ms windows, from −400 to 0 ms and from 200 to 600 ms from the appearance of the scene.

### Learning-related spatial selectivity

To quantify changes in the spatial selectivity across trials within a block, we calculated the proportion of variance explained (Ω^2^) by the chosen and the unchosen positions for each neuron over a 10-trial window collapsed across all blocks, sliding in 1 trial steps. The first window included the first 10 trials (trials from 1 to 10) of each block (e.g., 50 trials in the case of 5 total blocks recorded for a specific neuron), divided by chosen/unchosen position; the second window included the second 10 trials (trials from 2 to 11) and so on, for a total of 21 different windows (last window trials 21 to 30). To ensure an adequate number of trials to calculate the explained variance, only neurons with at least 5 trials per position, chosen and unchosen, were selected in the first half of the blocks (runs 1 to 3) and the second half of the blocks (runs 4 to 6), the same population used for decoding. A total of 111 neurons was selected for this analysis. This procedure was repeated averaging firing rate activity in bins of 250 ms, starting from 600 ms before the event of interest (scene appearance in the Delay period and peripheral target touch in the Touch PT period) until 800 ms later, moving in steps of 25 ms. The resulting heatmaps of each neuron were then averaged to obtain the heatmap of the whole population. The heatmaps were further corrected to account for possible task design bias. For each possible position of the correct target, the incorrect target was presented randomly at only 6 other possible positions (and not on the 9 possible positions, in a balanced and controlled fashion), excluding the position of the correct target and 3 other positions. A summary of the possible combinations for each position of the correct target is depicted in S6A Fig. For this reason, partial overlap could occur for some pairs of chosen/unchosen positions. An example is shown in S6B Fig. In the third session, Monkey 1 chose the target in position 4 in 57 trials; in 23 of these (40.4%) the unchosen target was presented in position 10 (S6B Fig, left). The same example is shown for trials of unchosen position 7 in the same session (S6B Fig, right). In this example, any neuron modulated by the chosen position 4/unchosen position 7 could show partial residual coding for the unchosen position 10/chosen position 1, respectively. For this reason, the percentage of explained variance shown in the heatmaps was corrected using the following procedure. For each neuron and each bin, the firing rate in each trial for each chosen position used to calculate the explained variance was replaced with the average firing rate calculated over all trials of the same condition. In this way, we artificially removed the variance within the groups that was possibly due to the paired unchosen position, raising the variance explained by the chosen positions to 100%. The trials were then grouped again by the unchosen position to calculate the residual variance explained due to possible bias in the pairing of positions. The value of the expected residual explained variance for the unchosen position in the case of perfect dissociation is 0%, while in the case of complete overlap is expected to be 100%. We found a mean residual explained variance around 13.8% across all bins for the unchosen position due to the unbalance of the trials. This procedure was also applied by grouping the trials by unchosen position to calculate the residual variance explained by the chosen position (mean residual explained variance around 14.0% across all bins). The original explained variance values obtained in the heatmaps were then reduced in proportion to the residual explained variance value obtained by this method. Significant bins were then identified by comparing the values of each bin with those of a null-model obtained by shuffling the conditions (1,000 shuffles, p value <0.001, Bonferroni corrected).

## Supporting information

S1 Table Comparison of reaction times between runs for Monkey 1 and Monkey 2 (two-sample Kolmogorov–Smirnov test). (DOCX)

S1 Fig Estimated location of the UTAH arrays on the lateral frontal pole of for both monkeys; 4 × 4 mm arrays were used for Monkey 1 (in turquoise) and 2.4 × 3.2 mm arrays were used for Monkey 2 (in gray). Reconstruction of locations was made based on pictures made during the surgeries. The coordinates of each array (center of the array) are the following: Monkey 1 right: 4.6 mm from midline/5.45 mm from the apex. Monkey 1 left: 6 mm from midline/5.45 mm from the apex. Monkey 2 right: 4 mm from midline/4.55 mm from the apex. Monkey 2 left: 3 mm from midline/2.65 mm from the apex. Note that the reconstruction is built on an average macaque brain [51] and not based on the MRI of each animal. Consequently, it is an approximation of the position of each array on an atlas template. For example, the left array of Monkey 1 was measured to be at 6 mm from the midline and 5.45 mm from the apex but did not reach the PS (probably due to the brain curvature not taken into account on these reconstructions).
(TIF)

S2 Fig Run selectivity from a 1-way ANOVA analysis. (A) Proportion of neurons (n = 56 neurons significant to the 1-way ANOVA, run as factor) with a highest or lowest firing rate in each run. The firing rate should have the highest or lowest value in a run and not necessarily be significantly higher or lower than the others. (B) Spike-density of the 56 significant neurons to the 1-way ANOVA around the delay period (left). Same representation for significant neurons with a preference in the first run and a higher (middle) or a lower (right) firing rate in the first run. Source data are available in S1 Data.
(TIF)

S3 Fig Same as Fig 4 only for Monkey 1. Neural activity is aligned at the presentation of the scene (start of Delay epoch). Blue bottom lines represent the periods during which classification accuracy is above chance level for at least 5 consecutive bins. n = number of neurons. Shaded gray areas represent the epoch during which the single neuron preferences were assessed (see Fig 2B). Source data are available in S1 Data.
(TIF)

S4 Fig Same as Fig 4 only for Monkey 2. Neural activity is aligned at the presentation of the scene (start of Delay epoch). Blue bottom lines represent the periods during which the classification accuracy is above chance level for at least 5 consecutive bins. n = number of neurons. Shaded gray areas represent the epoch during which the single neuron preferences were assessed (see Fig 2B). Source data are available in S1 Data.
(TIF)

S5 Fig Same as Fig 5 for Monkey 1 and Monkey 2, separately. Source data are available in S1 Data.
(TIF)

S6 Fig (A) Possible pairs of target locations; for each correct target positions (green circles), the possible positions of the incorrect target are shown (red circles). (B) Example of chosen/unchosen target pairing from the third session of Monkey 1. Left: the orange circles indicate how many of the 57 trials with the target chosen in position 4 (blue circle) were paired with unchosen positions 1, 3, 6, 7, 9, and 10. Red number is the most paired unchosen position with the chosen position 4 in this session. Right: same for the unchosen position 7 (45 trials).
(TIF)

S7 Fig Same as Fig 6A for Monkey 1 and Monkey 2, separately. Source data are available in S1 Data.
(TIF)

S8 Fig Same as Fig 6D for Monkey 1 and Monkey 2, separately. Source data are available in S1 Data.
(TIF)

S9 Fig Same as Fig 6D around the occurrence of the feedback. Source data are available in S1 Data.
(TIF)

S10 Fig Classification accuracy between trials in the second run in which the same object as in the first run was to be chosen (Previously Chosen trials) and those in which the object not chosen in the first run was to be chosen (Previously Unchosen trials). Left: all trials in the second run. Right: only correct trials in the second run. Neural activity is aligned at the presentation of the scene (start of Delay epoch). n = number of neurons. Source data are available in S1 Data.
(TIF)

S1 Data Source data. (XLSX)

## Abbreviations

CT: central target
FPC: frontopolar cortex
lFPC: lateral part of the frontopolar cortex
mFPC: medial part of the frontopolar cortex
OIP: object-in-place
