# Supplementary material for: Neurons in the monkey frontopolar cortex encode learning stage and goal during a fast learning task
Source: PLoS Biol. 2024 Feb 16;22(2):e3002500. doi: 10.1371/journal.pbio.3002500 (PMC10903959; doi:10.1371/journal.pbio.3002500)
Supplement: S1 Table — (DOCX) [file pbio.3002500.s001.docx]

| **Monkey1** | Run1 | Run2 | Run3 | Run4 | Run5 | Run6 |
| --- | --- | --- | --- | --- | --- | --- |
| Run1 | - | p<0.001 | p<0.001 | p<0.001 | p<0.001 | p<0.001 |
| Run2 | p<0.001 | - | ns | p<0.05 | ns | ns |
| Run3 | p<0.001 | ns | - | ns | ns | ns |
| Run4 | p<0.001 | p<0.05 | ns | - | ns | ns |
| Run5 | p<0.001 | ns | ns | ns | - | ns |
| Run6 | p<0.001 | ns | ns | ns | ns | - |

| **Monkey2** | Run1 | Run2 | Run3 | Run4 | Run5 | Run6 |
| --- | --- | --- | --- | --- | --- | --- |
| Run1 | - | p<0.001 | p<0.001 | p<0.001 | p<0.001 | p<0.001 |
| Run2 | p<0.001 | - | p<0.001 | p<0.05 | p<0.001 | p<0.001 |
| Run3 | p<0.001 | p<0.001 | - | p<0.001 | p<0.001 | p<0.001 |
| Run4 | p<0.001 | p<0.05 | p<0.001 | - | ns | ns |
| Run5 | p<0.001 | p<0.001 | p<0.001 | ns | - | ns |
| Run6 | p<0.001 | p<0.001 | p<0.001 | ns | ns | - |
