# Supplementary figures and images for: Neurons in the monkey frontopolar cortex encode learning stage and goal during a fast learning task
Source: PLoS Biol. 2024 Feb 16;22(2):e3002500. doi: 10.1371/journal.pbio.3002500 (PMC10903959; doi:10.1371/journal.pbio.3002500)

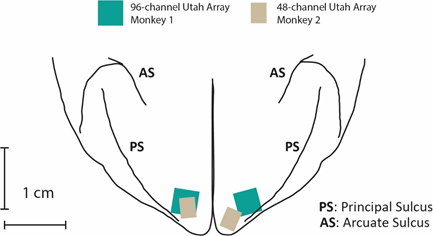

Supplement: S1 Fig — Reconstruction of locations was made based on pictures made during the surgeries. The coordinates of each array (center of the array) are the following: Monkey 1 right: 4.6 mm from midline/5.45 mm from the apex. Monkey 1 left: 6 mm from midline/5.45 mm from the apex. Monkey 2 right: 4 mm from midline/4.55 mm from the apex. Monkey 2 left: 3 mm from midline/2.65 mm from the apex. Note that the reconstruction is built on an average macaque brain [51] and not based on the MRI of each animal. Consequently, it is an approximation of the position of each array on an atlas template. For example, the left array of Monkey 1 was measured to be at 6 mm from the midline and 5.45 mm from the apex but did not reach the PS (probably due to the brain curvature not taken into account on these reconstructions). (TIF) [file pbio.3002500.s002.tif]

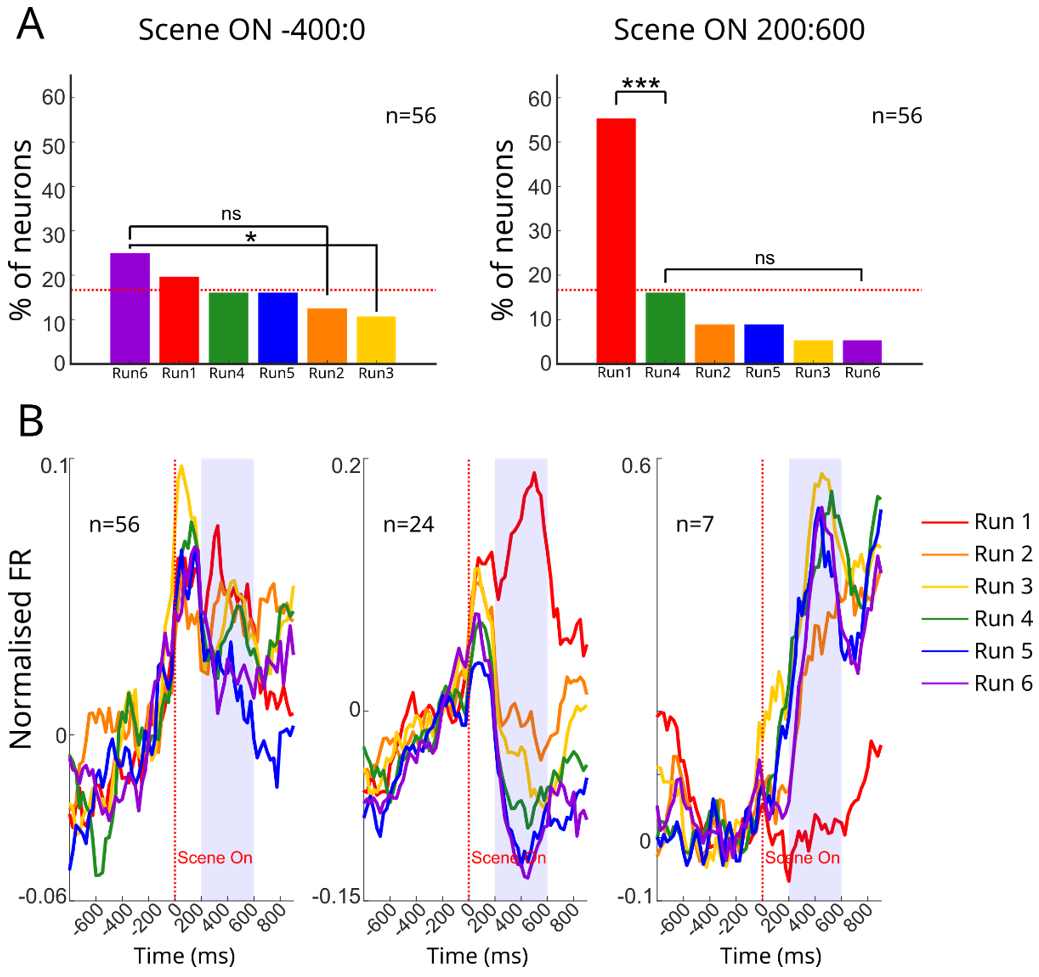

Supplement: S2 Fig — (A) Proportion of neurons (n = 56 neurons significant to the 1-way ANOVA, run as factor) with a highest or lowest firing rate in each run. The firing rate should have the highest or lowest value in a run and not necessarily be significantly higher or lower than the others. (B) Spike-density of the 56 significant neurons to the 1-way ANOVA around the delay period (left). Same representation for significant neurons with a preference in the first run and a higher (middle) or a lower (right) firing rate in the first run. Source data are available in S1 Data. (TIF) [file pbio.3002500.s003.tif]

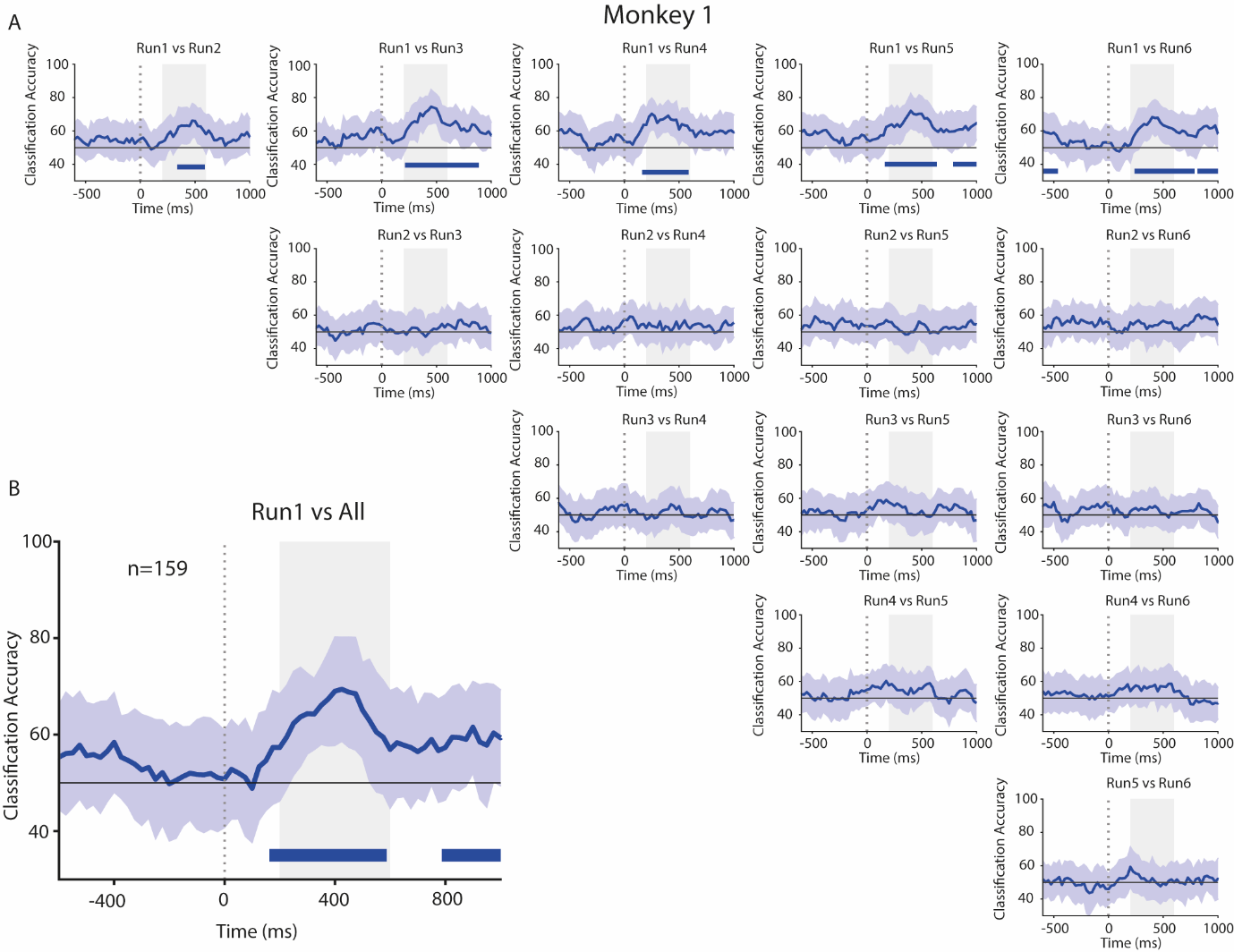

Supplement: S3 Fig — Neural activity is aligned at the presentation of the scene (start of Delay epoch). Blue bottom lines represent the periods during which classification accuracy is above chance level for at least 5 consecutive bins. n = number of neurons. Shaded gray areas represent the epoch during which the single neuron preferences were assessed (see Fig 2B). Source data are available in S1 Data. (TIF) [file pbio.3002500.s004.tif]

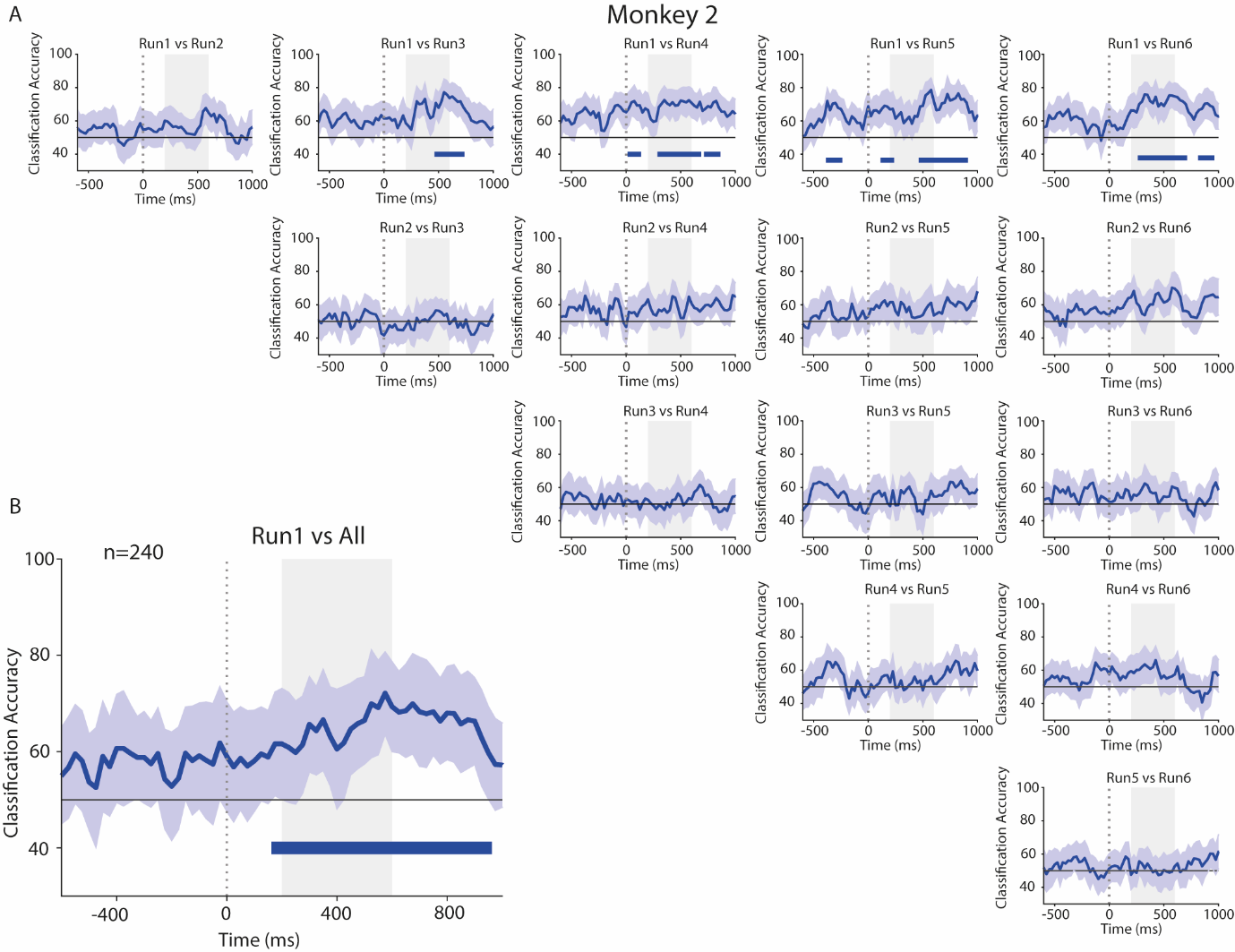

Supplement: S4 Fig — Neural activity is aligned at the presentation of the scene (start of Delay epoch). Blue bottom lines represent the periods during which the classification accuracy is above chance level for at least 5 consecutive bins. n = number of neurons. Shaded gray areas represent the epoch during which the single neuron preferences were assessed (see Fig 2B). Source data are available in S1 Data. (TIF) [file pbio.3002500.s005.tif]

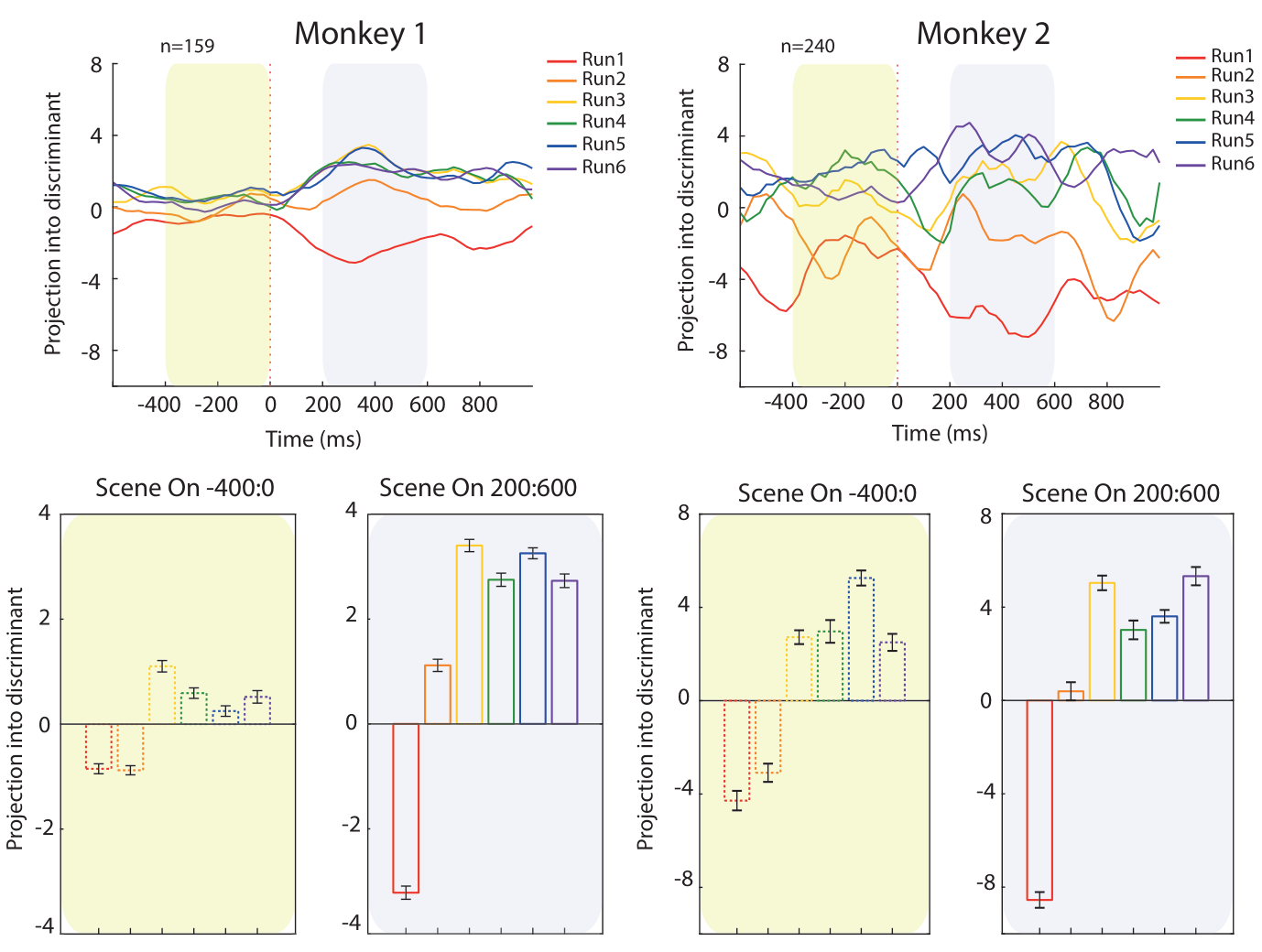

Supplement: S5 Fig — Source data are available in S1 Data. (TIF) [file pbio.3002500.s006.tif]

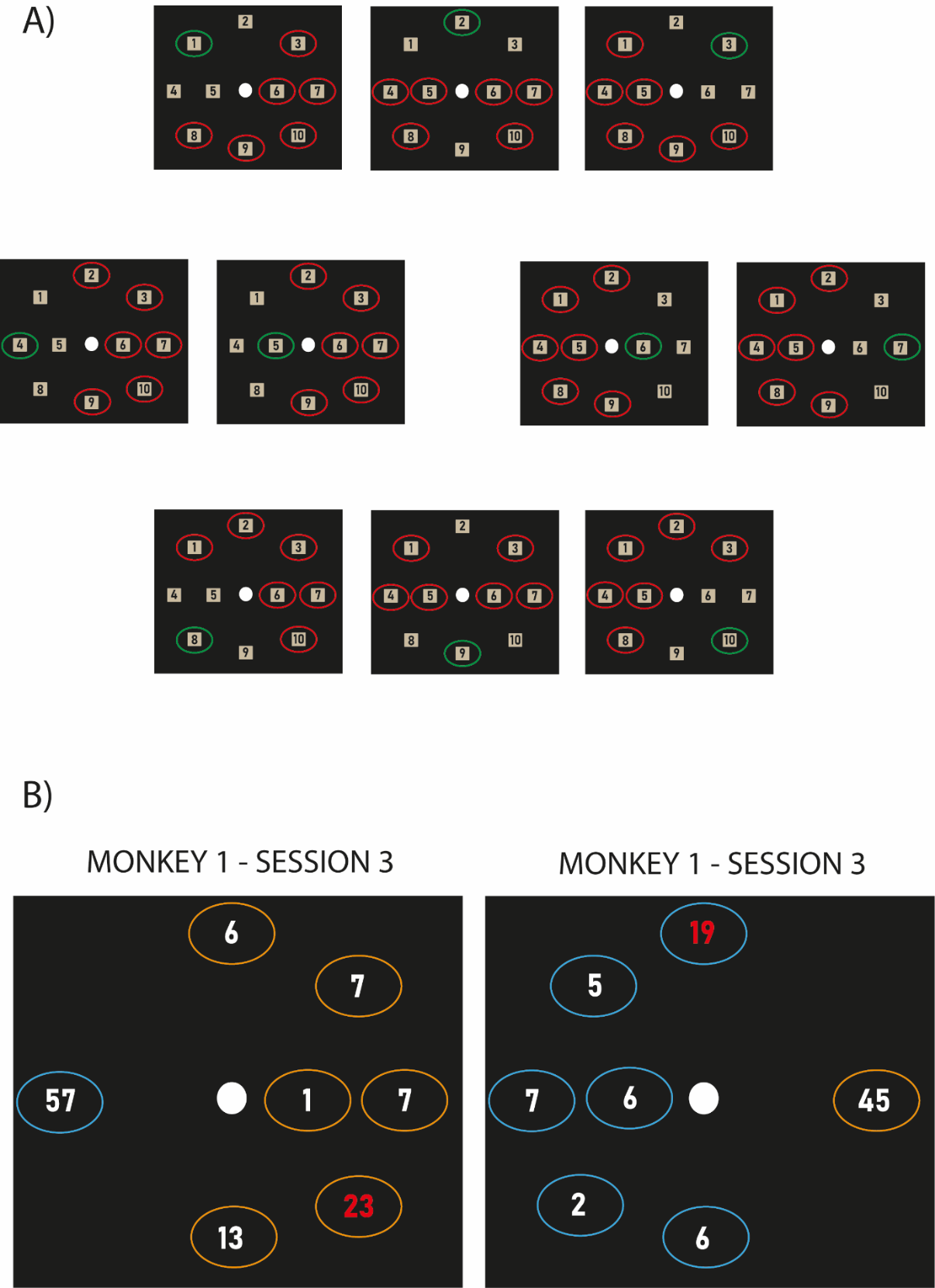

Supplement: S6 Fig — (A) Possible pairs of target locations; for each correct target positions (green circles), the possible positions of the incorrect target are shown (red circles). (B) Example of chosen/unchosen target pairing from the third session of Monkey 1. Left: the orange circles indicate how many of the 57 trials with the target chosen in position 4 (blue circle) were paired with unchosen positions 1, 3, 6, 7, 9, and 10. Red number is the most paired unchosen position with the chosen position 4 in this session. Right: same for the unchosen position 7 (45 trials). (TIF) [file pbio.3002500.s007.tif]

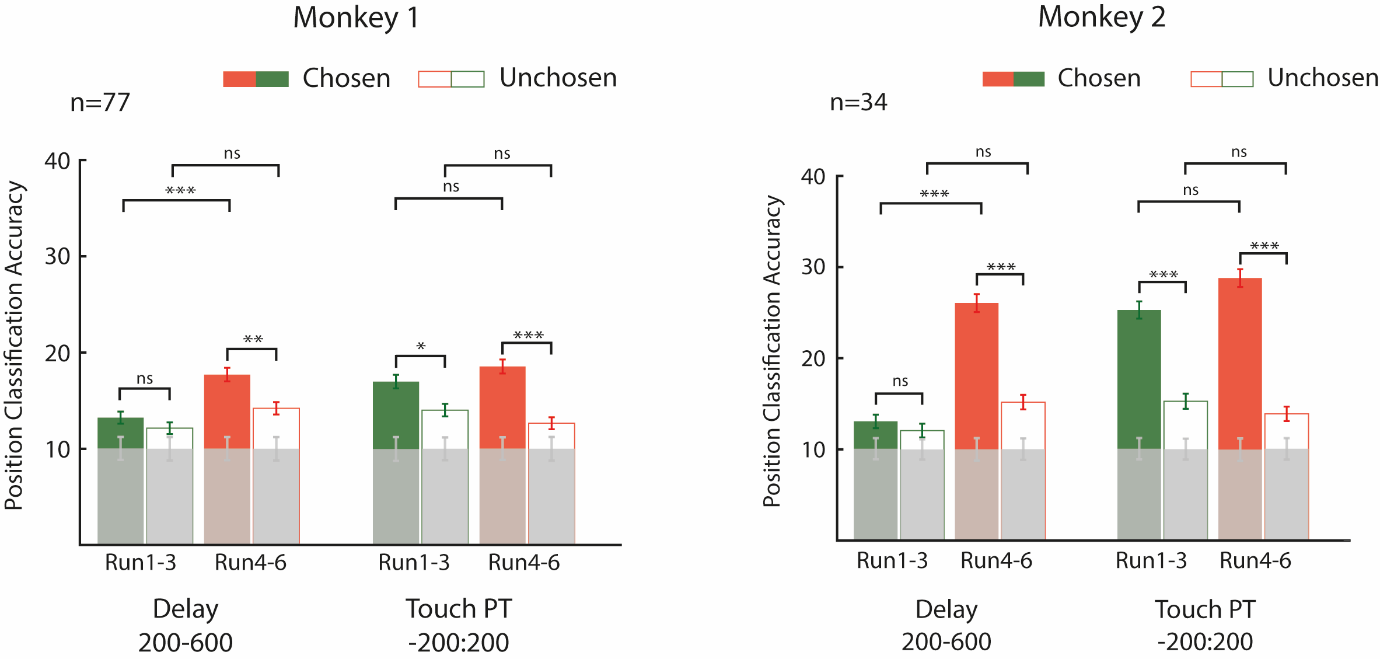

Supplement: S7 Fig — Source data are available in S1 Data. (TIF) [file pbio.3002500.s008.tif]

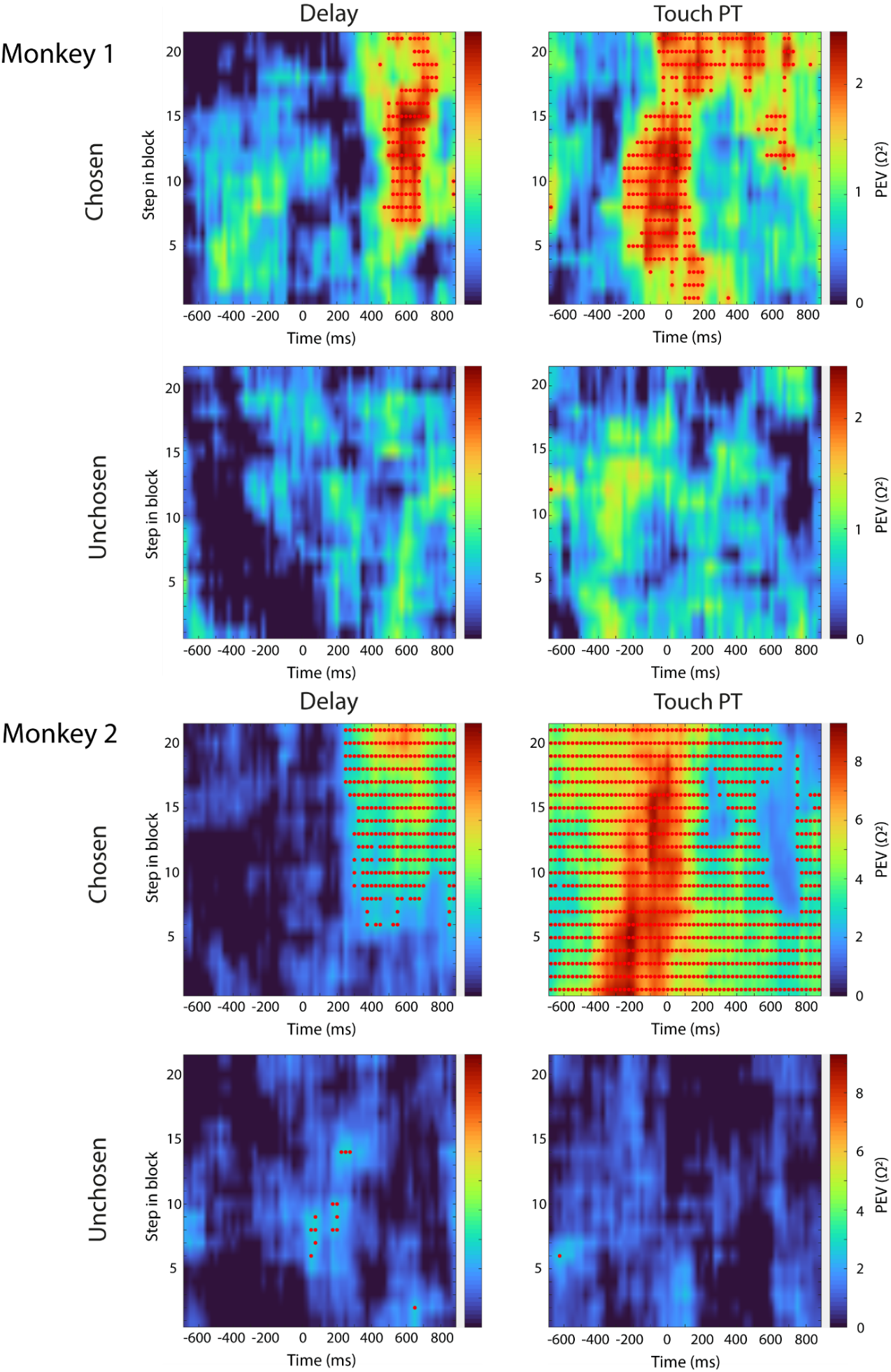

Supplement: S8 Fig — Source data are available in S1 Data. (TIF) [file pbio.3002500.s009.tif]

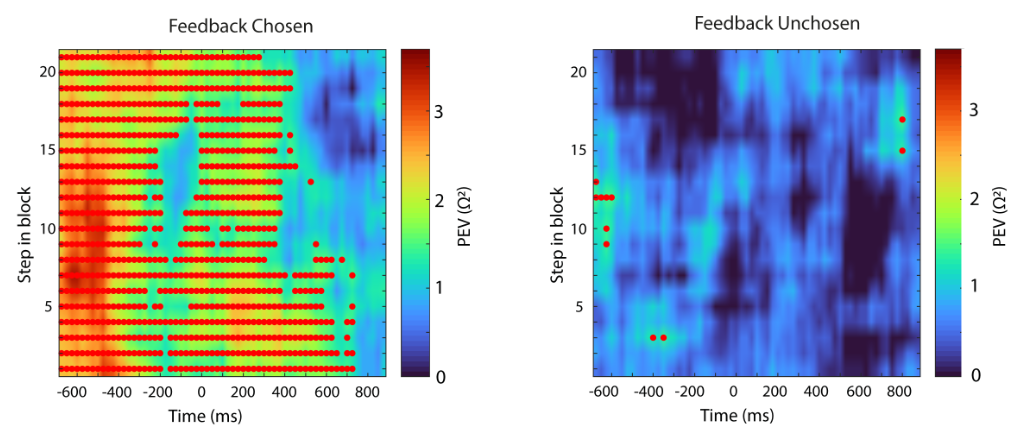

Supplement: S9 Fig — Source data are available in S1 Data. (TIF) [file pbio.3002500.s010.tif]

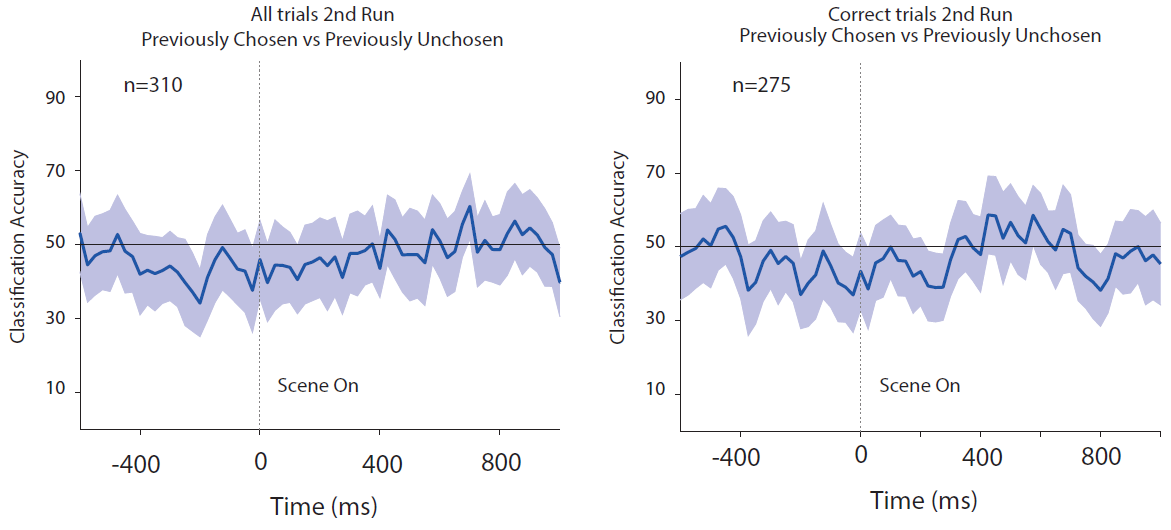

Supplement: S10 Fig — Left: all trials in the second run. Right: only correct trials in the second run. Neural activity is aligned at the presentation of the scene (start of Delay epoch). n = number of neurons. Source data are available in S1 Data. (TIF) [file pbio.3002500.s011.tif]
